# Evaluation of Incidence and Clinical Features of Antibody-Associated Autoimmune Encephalitis Mimicking Dementia

**DOI:** 10.1155/2014/935379

**Published:** 2014-03-17

**Authors:** Arzu Çoban, Cem İsmail Küçükali, Başar Bilgiç, Nazlı Yalçınkaya, Hazal Haytural, Canan Ulusoy, Selin Turan, Sibel Çakır, Alp Üçok, Hüseyin Ünübol, Hasmet A. Hanagasi, Hakan Gürvit, Erdem Tüzün

**Affiliations:** ^1^Department of Neurology, Istanbul Faculty of Medicine, Istanbul University, Millet Caddesi, Capa, 34390 Istanbul, Turkey; ^2^Department of Neuroscience, Institute for Experimental Medical Research (DETAE), Istanbul University, 34093 Istanbul, Turkey; ^3^Department of Psychiatry, Istanbul Faculty of Medicine, Istanbul University, 34390 Istanbul, Turkey; ^4^Istanbul Erenkoy Psychiatric and Neurological Disorders Hospital, 34736 Istanbul, Turkey

## Abstract

*Background*. Anti-neuronal autoimmunity may cause cognitive impairment that meets the criteria for dementia. *Objective*. Our aim was to detect the incidence and clinical features of autoimmune encephalitis imitating clinical findings of primary dementia disorders and to delineate the validity of anti-neuronal antibody screening in dementia patients. *Methods*. Fifty consecutive patients fulfilling the clinical criteria for primary dementia, 130 control patients, and 50 healthy controls were included. Their sera were investigated for several ion channel and glutamic acid decarboxylase (GAD) antibodies by a cell-based assay, radioimmunoassay, and ELISA, as required. *Results*. Sixteen patients satisfying dementia criteria had atypical findings or findings suggestive of autoimmune encephalitis. N-methyl-D-aspartate receptor (NMDAR) antibody was detected in a patient with dementia, Parkinsonism, and REM sleep behavior disorder (RBD) fulfilling the criteria for dementia with Lewy bodies (DLB). One control patient with bipolar disease displayed low anti-GAD antibody levels. *Conclusions*. Our study showed for the first time the presence of parkinsonism and RBD in an anti-NMDAR encephalitis patient mimicking DLB. Although autoimmune encephalitis patients may occasionally present with cognitive decline, most dementia patients do not exhibit anti-neuronal antibodies, suggesting that routine analysis of these antibodies in dementia is not mandatory, even though they display atypical features.

## 1. Introduction

Paraneoplastic or non-paraneoplastic limbic encephalitis is an autoimmune disorder, presenting with acute/subacute onset, monophasic disease course, and unique clinical findings. Alternatively, some limbic encephalitis cases may present with progressive dementia and behavioral symptoms mimicking chronic neurological or psychiatric disorders [1, 2], emphasizing the importance of setting clinical criteria for selection of patients that require anti-neuronal antibody screening. Reported clinical features suggesting autoimmune encephalitis include a subacute onset with a rapidly progressive, often fluctuating course and inflammatory cerebrospinal fluid findings [3].

In this study, to investigate whether primary dementia patients might display well-characterized antibodies associated with autoimmune encephalitis and to determine clinical features associated with dementia-like autoimmune encephalitis, we investigated various anti-neuronal antibodies in sera of patients fulfilling the criteria for primary dementia.

## 2. Methods

### 2.1. Patients

Among patients consecutively admitted to our outpatient clinic, 50 cases fulfilling the DSM-IV-TR criteria for primary dementia were included. These patients received the diagnosis of Alzheimer disease (AD), frontotemporal dementia (FTD), or dementia with Lewy bodies (DLB) (*n* = 1) according to the relevant international criteria [4–6]. Only patients with normal complete blood count, blood biochemistry analyses, thyroid function tests, sedimentation rate, vasculitic/rheumatological antibody screening, syphilis serology, and cranial MRI were included. Control cases included consecutive patients with chronic neurological (Parkinson disease) and psychiatric (bipolar disorder, schizophrenia) disorders fulfilling the relevant DSM-IV-TR criteria (*n* = 130) and healthy individuals (*n* = 50). Patients gave informed consent for the study, which had been approved by our local ethics committee. Data regarding medical history and demographic features were collected for each patient. Severity and staging of dementia were assessed by mini-mental state examination (MMSE) and global deterioration scale (GDS), respectively. In dementia patients, “atypical features” suggestive of autoimmune encephalitis were determined based on published guidelines and our observations [7]. Early onset (<40 years old), rapid progression (final MMSE-MMSE during diagnosis/disease duration >3.3) [8], subacute onset of dementia, atypical onset of dementia (primary progressive aphasia or posterior cortical atrophy), presence of seizures, CSF oligoclonal band (OCB) positivity, and/or pleocytosis and white matter lesions in the absence of vascular risk factors were accepted as atypical features.

### 2.2. Antibody Tests

Voltage-gated potassium channel (VGKC) complex antibodies (normal value < 100 pM) were identified by radioimmunoprecipitation using ^125^I-dendrotoxin-VGKC [1]. Antiglutamic acid decarboxylase (GAD) antibody (normal value < 10 U/mL) was investigated by commercial ELISA kits (Euroimmun, Luebeck, Germany) using standard protocols. The antibodies to N-methyl-D-aspartate receptor (NMDAR), *α*-amino-3-hydroxy-5-methyl-4-isoxazolepropionic acid receptor (AMPAR), VGKC-complex proteins contactin-associated protein-like 2 (CASPR2), and leucine-rich glioma inactivated 1 (LGI1) were investigated by a commercial kit using a cell-based assay utilizing transfected HEK293 cells (Euroimmun). Nontransfected HEK293 cells were used as negative controls. Positive controls of the assay kits and the archived sera that were previously shown to be antibody positive were used as positive controls.

## 3. Results

The study was performed in a group of 50 (24 women, 26 men) consecutive patients fulfilling the criteria for primary dementia (39 AD, 10 FTD, and 1 DLB) followed in our outpatient clinic. A control group included gender-matched 100 chronic psychiatric patients (45 women, 55 men; 50 bipolar disorder, 50 schizophrenia), 30 Parkinson disease patients (12 women, 18 men), and 50 healthy individuals (25 women, 25 men). The mean age of the patients with dementia was 72 ± 14 years (range: 25–89 years) and the mean disease duration was 7 ± 3 years (range: 1–15 years). The mean GDS was 5 ± 0.7 (range 4–7). The mean age of the control group with chronic psychiatric patients, PD patients, and healthy persons were, respectively, 32 ± 9 years (range: 19–63 years), 64 ± 8 years (range: 41–78 years), and 53 ± 16 years (range: 22–86 years). Mean disease durations of chronic psychiatric and PD patients were 4 ± 1 years (range: 1–5 years) and 8 ± 4 years (range: 2–12 years), respectively. The routine laboratory tests of dementia patients and controls showed no evident abnormalities. None of the patients had autoimmune disease or cancer. Sixteen dementia patients (11 AD, 4 FTD, and 1 DLB) displayed at least one of the determined atypical features for dementia (Table 1).

The NMDAR antibody was positive in one patient fulfilling the DLB criteria (Case 1). Another patient with bipolar disease (Case 2) had low (35.4 U/mL) GAD antibody levels. No antibodies were detected in sera of other cases.

*Case 1*. This 58-year-old man presented with a 4-month history of amnesia, aggressive behavior, visual hallucinations, urinary incontinence, and clinically diagnosed rapid eye movement (REM) behavior disorder (RBD) fluctuating from hour to hour. His wife reported acting out violent dreams, vocalization, and episodes of kicking, biting, and screaming during sleep consistent with RBD occurring every 1 or 2 times every night. A polysomnogram could not be performed.

Neurological examination revealed disorientation in time and place, positive glabellar tap, rigidity, and bradykinesia. The patient demonstrated a stooped posture and festinant gait without retropulsion. He did not display tremor and did not have a history of treatment with any medication including antipsychotics. The patient scored 13/30 on MMSE and 4 on GDS. Detailed neuropsychological examination showed moderately impaired attention, verbal memory, visual memory, and visual-spatial perception. Routine blood tests and T1-, T2-, FLAIR-, and diffusion-weighted MRIs were normal. EEG showed widespread slow waves. CSF cell count, protein concentration, and 14-3-3 protein levels were normal and no OCB were detected. Serum NMDAR antibody was found positive, whereas other investigated antibodies were negative. The atypical features included subacute onset of dementia and rapid progression (Table 1). His symptoms promptly regressed under pulse steroid (1000 mg/day iv for 5 days) and intravenous immunoglobulin (IVIG) (30 gr total for 5 days) treatments. One month after steroid and IVIG introduction, MMSE had improved to 22/30, disorientation had partially improved, and visual hallucinations had disappeared. He subsequently received monthly pulse steroid and IVIG treatments and his symptoms completely disappeared in 3 months.

*Case 2*. The GAD-antibody positive bipolar disease patient was a 31-year-old woman with disease duration of 3 years. She had typical manic and depressive episodes lasting from a few days to weeks with no psychotic features. During blood sampling for antibody testing, she had mild depressive symptoms under treatment. Her medical history did not suggest subacute onset or rapid progression and her MRI, EEG, and neuropsychological examinations were normal.

## 4. Discussion

The key finding of our study was the clinical presentation of NMDAR antibody-associated autoimmune encephalitis masquerading as DLB, demonstrating the potential for misdiagnosis of a reversible condition as a progressive dementia syndrome. Cognitive impairment has been reported in association with several autoantibodies directed against neuronal antigens such as VGKC-complex [1, 2], NMDAR [3, 9], and GAD [10, 11]. Anti-NMDAR encephalitis is particularly associated with psychiatric and neurological symptoms such as memory problems, seizures, hallucinations, and delusions [3]. Encephalitis patients with IgA NMDAR-antibodies may also present with slow cognitive impairment imitating dementia [9]. Also, NMDAR antibodies may lead to extrapyramidal symptoms such as dyskinesia, chorea, ballismus, and dystonia [3, 12]. However, to our knowledge, Parkinsonism-like symptoms have never been associated with anti-NMDAR encephalitis. Catatonia, a common finding in anti-NMDAR encephalitis [3], might be misinterpreted as Parkinsonism. However, the classical signs of Parkinsonism were observed in our patient, arguing against this possibility. Moreover, normal MRI and CSF examinations contradicted the diagnosis of anti-NMDAR encephalitis in our patient. On the other hand, major symptoms of our patient including memory impairment, visual hallucinations, fluctuation of symptoms, and Parkinsonism are core clinical features of DLB [5]. RBD, urinary incontinence, and EEG slow waves are occasionally encountered in both DLB and encephalitis patients further complicating the differential diagnosis [13, 14].

Among clinical features that were tested for prediction of autoimmune encephalitis, subacute onset of dementia and rapid progression were present in our patient, suggesting that dementia patients with these features might be screened for anti-neuronal antibodies. Presumably, dementia patients presenting with multisystem neurological involvement (e.g., multiple system atrophy), such as our patient, might also be considered as candidates for anti-neuronal antibody screening. Patients presenting with other features, which we assumed that they could be associated with autoimmune encephalitis (presenile onset, seizures, white matter lesions, and atypical onset and inflammatory CSF findings), were negative for anti-neuronal antibodies suggesting that these features do not predict autoimmune encephalitis. Also, the low seropositivity rate in our study argues against the screening of anti-neuronal antibodies in classical primary dementia patients. Nevertheless, investigation of a broader panel of antibodies (e.g., IgA anti-NMDAR) in future studies might yield additional seropositive patients validating such a screening.

Behavioral and cognitive impairments may also accompany anti-GAD-related neurological disorders. Previous studies have revealed that patients with both high (more than 2000 U/mL) and low (<100 U/mL) anti-GAD antibody levels may present with limbic symptoms [10, 11]. Similarly, we identified a bipolar disorder patient with low anti-GAD antibody levels and no atypical features suggestive of autoimmune encephalitis. Such low anti-GAD antibody levels have also been found in autoimmune neurological disorders such as myasthenia gravis and multiple sclerosis [15], suggesting that although low-level GAD antibody might presumably be nonpathogenic, its presence might be an indicator of autoimmunity. Whether our result is coincidental or autoimmunity might lead to mood disorders need to be further investigated.

In summary, our findings have several important implications. (1) Routine anti-neuronal antibody screening in classical primary dementia patients does not appear to be warranted. (2) Anti-NMDAR antibodies may be found in patients meeting the criteria for DLB. (3) Parkinsonism and RBD may be observed in anti-NMDAR encephalitis. (4) Early initiation of immunotherapy in these patients can produce an immediate recovery of cognitive function and independent living.

## Figures and Tables

**Table 1:** Dementia patients with atypical features that were presumed to be suggestive of autoimmune encephalitis.

	AD (*n* = 39)	FTD (*n* = 10)	DLB (*n* = 1)
Presenile onset of dementia	1 (2.5%)	2 (20%)	0 (0%)
Rapid progression	5 (12.8%)	3 (30%)	1 (100%)
Subacute onset of dementia	0 (0%)	0 (0%)	1 (100%)
Atypical onset of dementia (PPA or PCA)	1 (2.5%)	1 (10%)	0 (0%)
Seizures	6 (15.3%)	3 (30%)	0 (0%)
Positive OCB and/or CSF pleocytosis*	1 (2.5%)	2 (20%)	0 (0%)
Multiple white matter lesions without a vascular risk factor	1 (2.5%)	1 (10%)	0 (0%)

AD: Alzheimer disease; FTD: frontotemporal dementia; DLB: dementia with Lewy body; PPA: primary progressive aphasia; PCA: posterior cortical atrophy; OCB: oligoclonal band; CSF: cerebrospinal fluid.

*CSF examination was performed only in 7 dementia patients.
